# Oral Dimensions Related to Bit Size in Adult Horses and Ponies

**DOI:** 10.3389/fvets.2022.879048

**Published:** 2022-05-12

**Authors:** Mirjami Anttila, Marja Raekallio, Anna Valros

**Affiliations:** ^1^Department of Equine and Small Animal Medicine, Faculty of Veterinary Medicine, University of Helsinki, Helsinki, Finland; ^2^Department of Production Animal Medicine, Research Center for Animal Welfare, University of Helsinki, Helsinki, Finland

**Keywords:** animal welfare, bit fit, bit size, bridle, horse, mouth, oral dimensions

## Abstract

A bit that fits is essential for horse welfare and good communication with the ridden, driven or led horses. The bit causes pressure on the sensitive structures of the horse mouth. The aim of this study was to investigate variation in oral dimensions related to bit fit in adult horses and ponies and to evaluate bit fit by comparing oral dimensions with the currently used bit size selected by the horse owner. The study population consisted of 554 horses and ponies, 308 geldings and 246 mares, age 5-29 years, presented for routine dental care. Oral dimensions: mouth width, distance between upper and lower jaw, tongue thickness and lower jaw width, were measured under sedation. Oral dimensions were compared with the most used bit mouthpiece size presented to the researcher by the owner. Bit type and material were recorded. All oral dimensions in adult horses and ponies varied by breed and sex. Mouth width and distance between upper and lower jaw correlated positively with age. Oral dimensions were significantly smaller in mares than in geldings. In coldblood Finnhorses, oral dimensions were greater than in other breeds; in ponies they were smaller. The majority of the oral dimensions correlated positively with each other. Lower jaw width did not correlate with tongue thickness. It was common to use a bit that did not fit the horse: the bit was either too short or too long (over 10 mm longer) compared to mouth width, compressed the tongue in between the upper and lower jaw, or the center link was of similar length compared to lower jaw width, thus possibly causing pressure points or a nutcracker effect on the bars of the lower jaw. Horses had, on average, space for a 14 mm thick bit without compressing the tongue. The results of this study can aid in choosing a horse bit size that fits correctly and does not cause discomfort. It is recommended that the fit of the bit is evaluated regularly as the horse ages.

## Introduction

A bit and bridle that fit are essential for preventing oral discomfort and ensuring good communication with the ridden, driven or led horses (1, 2). Maintaining good oral health is important for overall welfare of the horse (1, 3). Bit fitting means choosing a correct bit size and bit properties (design and material) and adjusting the location of the bit within the oral cavity with bridle cheek straps (4–6). The bit causes pressure on the sensitive structures of the horse mouth: on the lip commissures, buccal mucosa, tongue and the bars of the lower jaw (5–7), and depending on the bit type can also cause pressure to the hard palate and the base of the second premolars (5, 8, 9). Pressure (p) caused by the bit, *p* = Force (F)/Area (A), is related to the weight of the bit mouthpiece, cheekpiece tension, rein tension applied via reins, and to the contact area of the bit with the oral structures (3, 10). The noseband or other accessory equipment may affect rein tension, and thus the bit pressure (11). A bit that does not fit can potentially cause too much pressure, pinching or rubbing of oral tissues (12) or restrict tongue movement (7). An ill-fitting or inexpertly used bit can cause oral trauma (13–17) and thus discomfort or pain for the horse (3, 18). Oral discomfort can lead to undesirable behavior such as avoiding the bit pressure by opening the mouth and protruding the tongue, or resisting bit pressure by grasping the bit between the teeth (1, 4, 7). Bit acceptance by the horse is one of the evaluation points in dressage riding (19) and thus a bit that fits and its skillful use are prerequisites for good performance in dressage competition (7).

Location of the bit is restricted by the anatomical landmarks of the oral cavity (20). The bit lies on top of the tongue between the upper and lower jaw, taking space from the tongue (4). The horse's tongue is strong muscular tissue covered with a tough mucosa, and the dorsal part is strengthened by cartilage (21). The size of the tongue has been suggested to vary between horses (7, 8). The tongue works as a cushion to protect the bars of the lower jaw from the bit pressure (6, 20). The bony palatine arch and the bars of the lower jaw, covered with a thin 1–2 mm mucosa, limit the space for the bit and tongue in the dorsoventral direction (20). The incisors and second premolar teeth together with the lip commissures limit the location of the bit in the rostro-caudal direction (20). The bit is mobile inside the mouth (5, 10). Within the anatomical limitations, a horse can move the bit with its tongue and lips, and the rider or driver can move it by exerting rein tension, thus affecting the bit-tongue angle (10) and bit pressure distribution inside the mouth (5–7, 9, 20).

Previous studies related to bit and bridle fit have focused on bit position and action in the mouth (4–7, 9, 10, 22) and have described oral trauma caused by the bit (13–17). However, one study with 72 cavader horse heads reported considerable variation in oral dimensions between horses with respect to position and size of a snaffle bit (20). In another study hard palate depth was measured in 52 sedated horses and hard palate depth was found to vary from 5 to 27 mm, but no association with age, breed or sex was found (23). In craniometric studies with cadaver horses, the greatest variability in skull dimensions was detected in the nasal part of the skull (24, 25). Arabian horses had significantly shorter nasal length (mean 230 mm) compared to Thoroughbred (250 mm) and Standardbred (mean 280 mm), and Thoroughbred shorter than Standardbred (24). Another study in 14 adult Przewalski horses found geldings and stallions commonly to have higher skull dimensions compared with mares (26). In a radiographic study, no relationship between wither height and size of the oral cavity measured in head radiographs from eight horses was found (27). Craniometric and radiographic studies, however, focus on the dimensions of the bony parts of the skull and do not include soft tissues, which also affect the oral dimensions related to bit fit.

The purpose of the present study was to provide data to aid in choosing a correct bit size for each horse to avoid discomfort caused by an ill-fitting bit. We investigated variation in oral dimensions related to bit fit in adult horses and ponies and evaluated the fit of the bit by comparing oral dimensions with the currently used bit size selected by the horse owner. No similar measurements have been reported in living horses. The first hypothesis was that oral dimensions vary in horses and ponies. The second hypothesis was that oral dimensions are related to age, sex and breed. Therefore, if horse owners are aware of this relationship, it will affect their selection of bit size. Furthermore, the third hypothesis was that not all horses have a bit size, selected by the horse owner, that fits in relation to oral dimensions.

## Materials and Methods

### Horses

The study population consisted of 554 horses and ponies, 308 geldings and 246 mares ≥ 5 years of age (range 5-29, mean 11.7, SD 4.7 years) presented to routine dental care. Stallions were excluded because of their lower number. The breed distribution was 174 warmbloods (WB), 145 Finnhorses, 80 non-registered/breed crosses, 44 Standardbred (STB), 50 ponies (Shetland, Welsh, Connemara, riding pony) and 61 horses of other breeds (<23 horses/breed, the most common breeds being Estonian, Irish Cob, Quarter and Arab). A total of 274 horses were used for competitive riding, 218 were pleasure horses used for riding and/or driving, and 62 were used for harness racing. Data were collected between first of June 2016 and 31st of December 2020 at Savo Animal Hospital Ltd, Oulu Equine Clinic Ltd and the University of Helsinki, Equine Hospital, in Finland. All horses were examined by the same veterinarian experienced in equine dentistry (first author).

### Oral Dimensions

**Figure 1:**
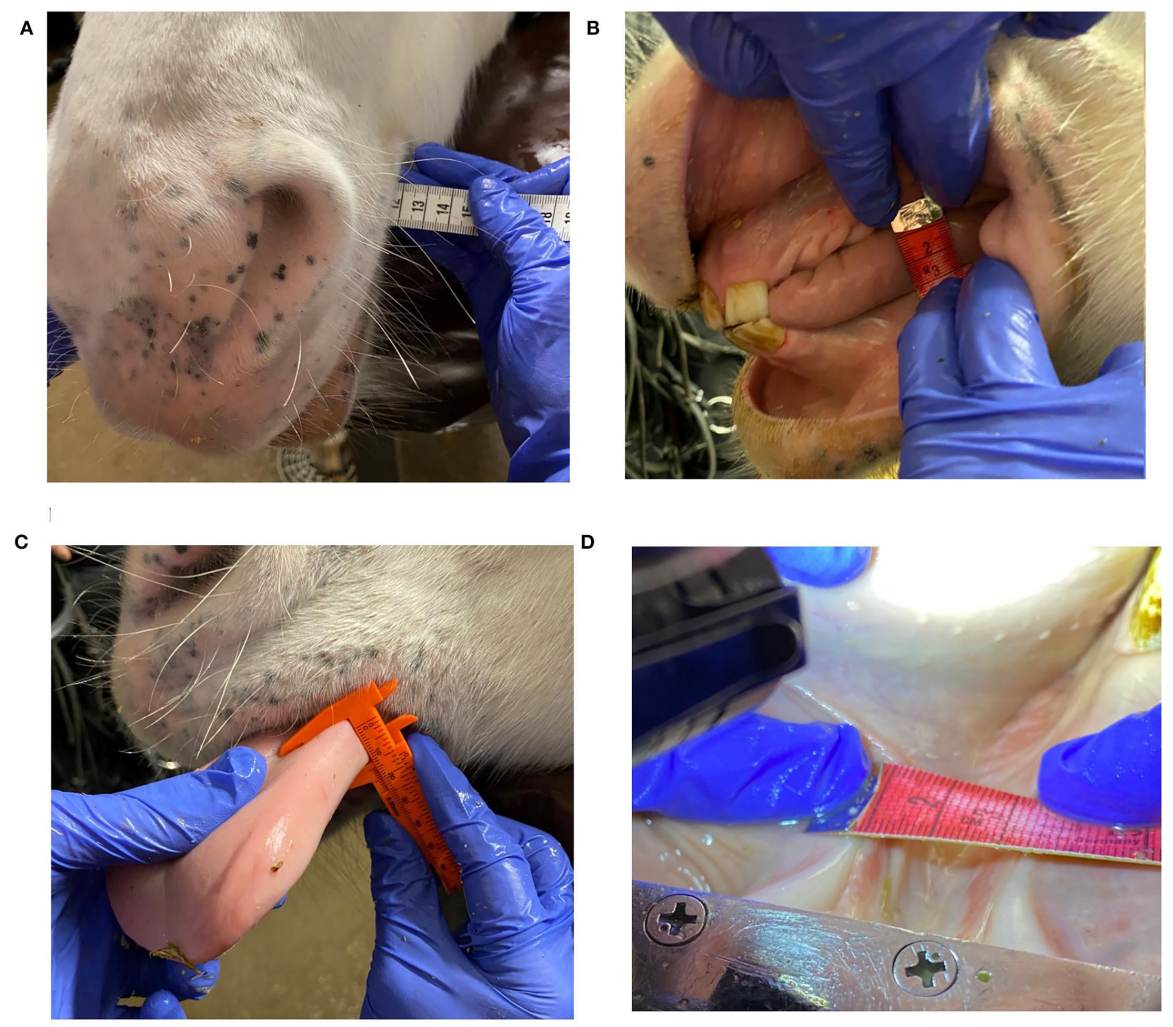
Oral dimension measurements: **(A)** mouth width, **(B)** distance between upper and lower jaw, **(C)** tongue thickness, **(D)** lower jaw width.

Oral examination was performed and oral dimensions were measured under sedation with detomidine 0.01–0.02 mg/kg intravenous (IV) (Domosedan vet 10 mg/ml, Orion Corporation, Finland) and butorphanol 0.01–0.02 mg/kg IV (Butordol 10 mg/ml, Intervet International, Germany). Sedation was supplemented if needed to complete the examination. Oral examination and dental care lasted 30-60 min. After dental treatment oral dimensions and bit size were measured with a flexible tape measure (Prym color Plus 150 cm) and a plastic sliding breadth caliper (Biltema, product number: 16-114) with an accuracy of 1 mm (Table 1; Figures 1A–D). Later in 2020, measurement of tongue thickness (*N* = 79) with a plastic sliding breadth caliper (Biltema, product number: 16-114) was included in the study (Figure 1C).

**Table 1:** Definitions of horse oral dimensions by anatomical landmarks.

**Variable**	**Definition**
Mouth width	Distance between left and right commissures of lips
Distance between upper and lower jaw	Distance between upper and lower jaw at level of interdental space at lip commissures when mouth is closed
Lower jaw width	Distance between middle dorsal surface of bars of lower jaw at lip commissures
Tongue thickness	Tongue thickness measured with caliper at lip commissures

### Bit Measurements

**Figure 2:**
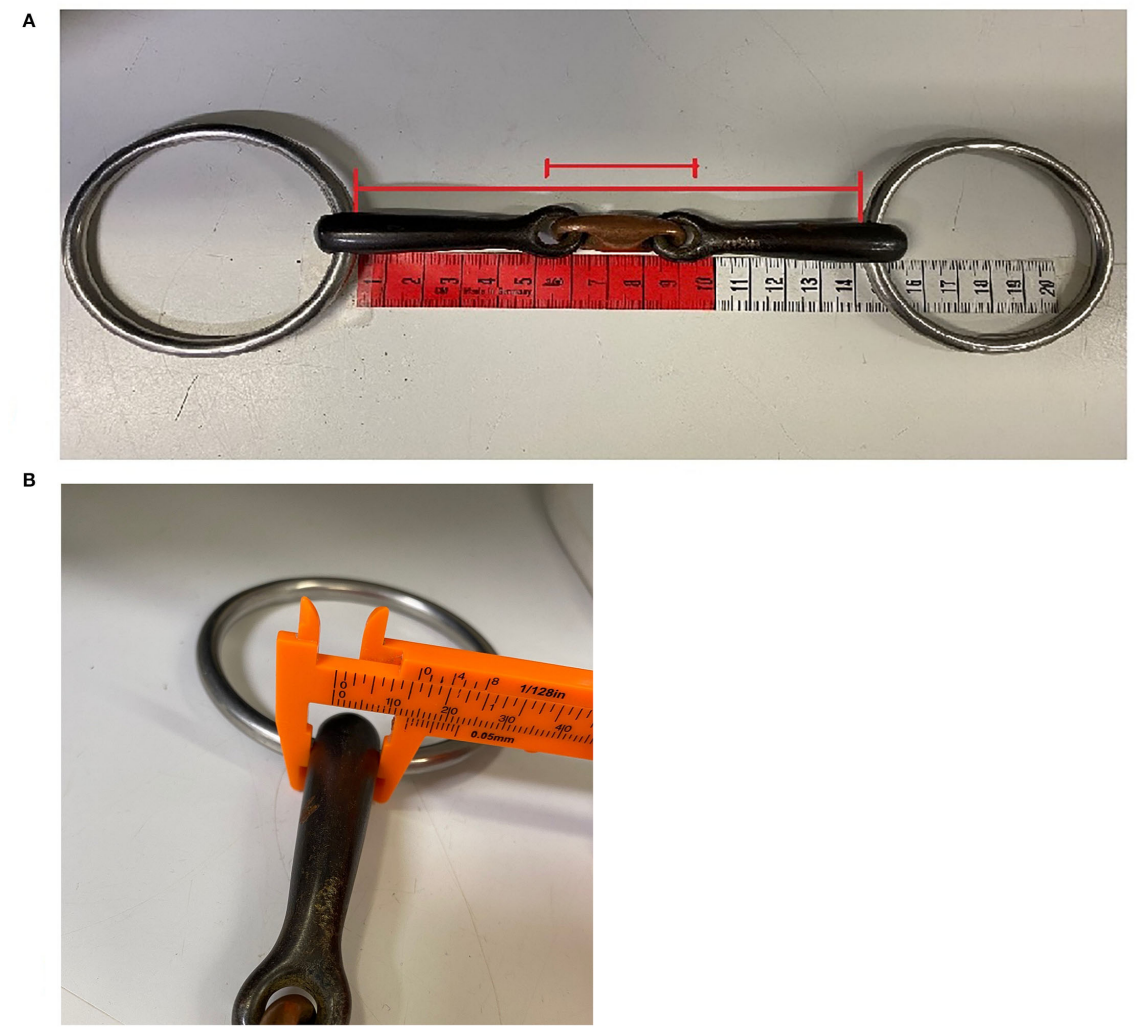
Bit size measurements: **(A)** bit mouthpiece and center link length, **(B)** bit mouthpiece thickness.

Horse owners were asked to bring the bits and bridles they used most commonly, if they wanted to have bit fitting instructions as part of the oral examinations and dental care. The most often used bit was included in the statistical analysis. Bit mouthpiece type, ring and material were recorded, and bit mouthpiece size was measured (Table 2; Figures 2A,B). Bit fit evaluation was done by calculating the difference between the corresponding oral and bit dimensions (Table 2).

**Table 2:** Definitions of bit size and bit fit.

**Variable**	**Definition**
Bit mouthpiece length	Distance between inner border of bit ring hole measured along caudal curve of bit mouthpiece
Bit mouthpiece thickness	Thickness/diameter (height) of bit mouthpiece between upper and lower jaw measured next to inner bit ring hole
Bit mouthpiece center link length	Length of bit mouthpiece center link in double-jointed or barrel bit
Bit space	Difference in distance between upper and lower jaw and tongue thickness
Tongue space	Difference in distance between upper and lower jaw and thickness of the bit
Bit length fit	Difference in bit mouthpiece length and mouth width—bit length fits, if bit mouthpiece length equal to or 1–10 mm longer than mouth width
Bit thickness fit	Difference in bit space and bit mouthpiece thickness – bit thickness fits, if bit mouthpiece thickness equal to or smaller than bit space
Bit center link length fit	Difference in bit center link length and lower jaw width—bit center link length fits, if center link length shorter or longer than lower jaw width

### Statistical Analysis

Statistical analyses were performed using IBM Analytics, SPSS Statistics (version 27.0). Separate linear regression models were performed for each measured oral dimension variable, and measured bit size variable. Linear regression models of each measured variable were used to analyse the effect of sex, breed and age. Warmblood, Standardbred, Finnhorse and pony mares and geldings were included in the study model. Variables were chosen based on preliminary univariate analysis and tested for normality. Age was taken as a covariate if significant correlation with the analyzed variable and age was found. The model residuals were assessed for normal distribution. *Post-hoc* pairwise comparisons were performed with Bonferroni adjustment. A paired sample *t*-test was used for testing differences between the right and left side in the distance between the upper and lower jaw. Variables related to bit fit in mares and geldings were compared with the *t*-test. The limit of significance was taken as *p* < 0.05.

## Results

### Study Population

All 554 horses and ponies were included in the final study population. Breed crosses with <23 horses/breed were excluded from the linear regression models and included in the descriptive analysis. None of the horse breeds were over-represented in either of the sexes (Pearson's chi-square = 6.90, degree of freedom (df) = 4, *p* = 0.142).

### Oral Dimensions

**Figure 3:**
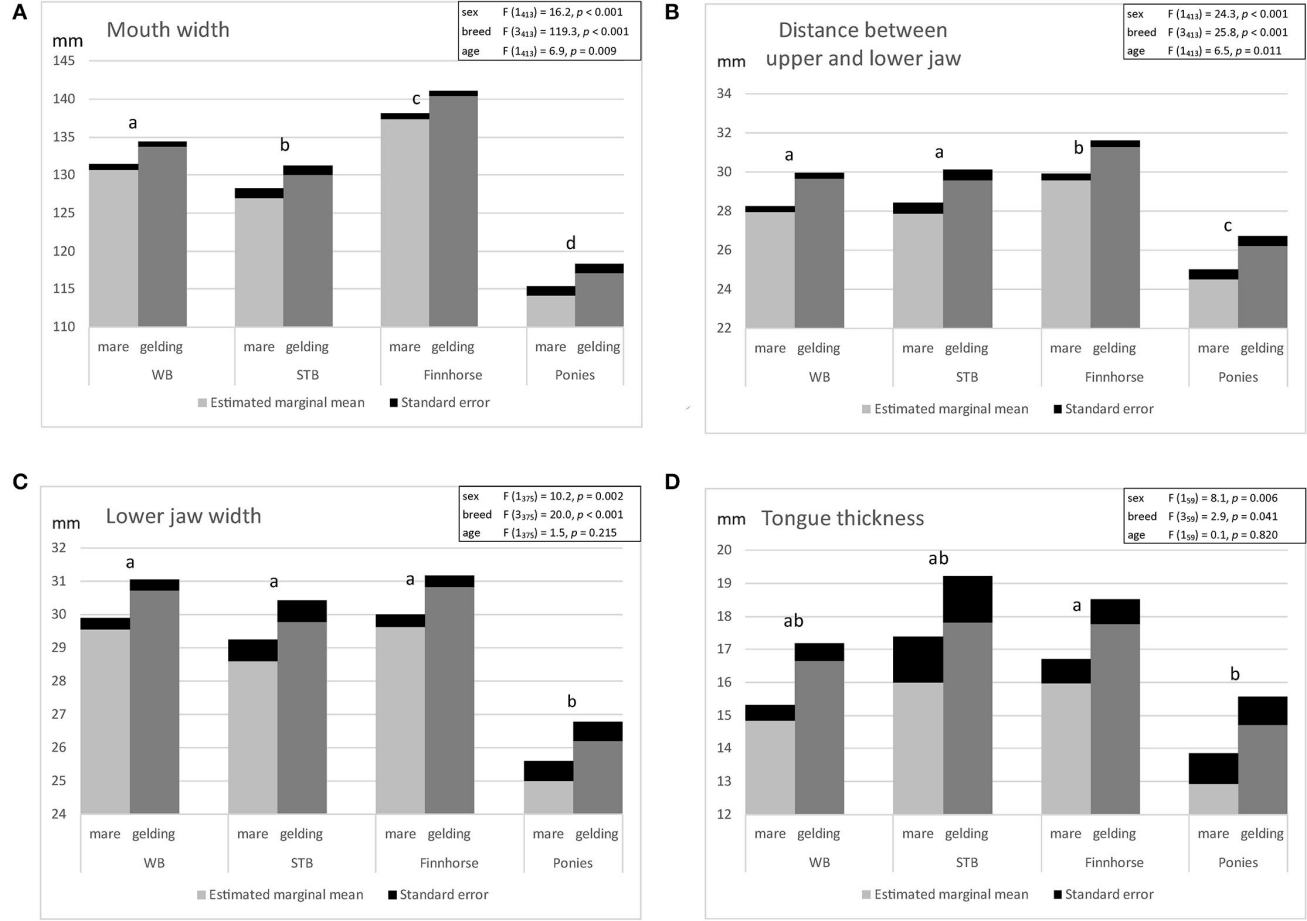
Estimated marginal means of linear regression model of oral dimensions in mares and geldings of different breeds [Warmblood (WB), Standardbred (STB), Finnhorse, ponies] of adult horses and ponies ≥ 5 years: **(A)** mouth width, **(B)** distance between upper and lower jaw, **(C)** lower jaw width, **(D)** tongue thickness (significant differences between breeds marked by differing letters).

The variation in oral dimensions in all horses and ponies in the study (*N* = 554) are presented in Table 3. No significant difference was detected between the right and left side in the distance between the upper and lower jaw (*t* = 0.65, *p* = 0.517), thus the mean value of both sides was taken for further calculations. Estimated marginal means of linear regression models of oral dimensions (*N* = 413) are presented in Figure 3. All oral dimensions were significantly smaller in mares than in geldings. All oral dimensions were associated with sex and breed, but mouth width and distance between upper and lower jaw correlated also with age (Figures 3A–D). Comparison of oral dimensions (Figures 3A–D) detected differences in mouth width between all breeds (*p* < 0.001). Finnhorses had all oral dimensions greater than other breeds, and all pony oral dimension measurements were the smallest. Other breeds differed in distance between upper and lower jaw, exceptWB and STB.

**Table 3:** Oral dimensions, bit size and bit fit of mares and geldings of different horse and pony breeds ≥ 5 years.

**Oral dimensions**	***N***	**Minimum (mm)**	**Maximum (mm)**	**Mean (mm)**	**Standard deviation**
Mouth width	554	95	165	131.3	10.3
Distance between upper and lower jaw	554	20	46	28.9	3.9
Lower jaw width	508	15	45	29.5	3.9
Tongue thickness	79	9	22	15.8	2.5
**Bit measurements**					
Bit length	422	100	160	135.2	10.7
Bit thickness	419	10	25	15.1	2.9
Bit center link length	220	20	65	35.6	7.3
**Bit fit**					
Bit space	79	5	22	14.0	3.2
Tongue space	419	0	36	13.7	4.7
Bit length fit	422	−20	30	3.6	7.2
Bit thickness fit	43	−9	12	0.3	3.5
Bit center link length fit	215	−15	40	5.9	8.5

Linear regression models revealed that breed, sex and age explain 47.7% of the variation in mouth width, 21.7% of the variation in tongue thickness, 20.1% of the variation in distance between the upper and lower jaw, and 16.7% of the variation in lower jaw width. Lower jaw width did not correlate with tongue thickness (*p* = 0.113). However, all other oral dimensions correlated positively with each other (*p* < 0.05). Correlations between oral dimensions are presented in the Supplementary Table 1.

### Bit Type

The bit mouthpiece type was recorded for 465/554 horses and the bit ring type and material for 454/554 horses. Only one bit was used in 268/465 (57.6%) horses, two different bits in 147/465 (31.6%) horses, three bits in 45/465 (9.7%) horses and 5/465 (1.1%) horses used a bitless bridle. The most used bit mouthpiece types were double-jointed snaffle (214/465, 46.0%), single-jointed snaffle (142/465, 30.5%), straight solid or flexible bit (54/465, 11.6%), barrel bit (30/465, 6.5%), straight ported bit (9/465, 1.9%) and other type of bit (11/465, 2.4%). The most common bit cheek type was loose O-ring (300/454, 66.1%). Other bit cheek types were fixed ring (eggbutt/D-ring, full-/half-cheek) (116/454, 25.6%), vertical shank (Weymouth, Pelham, Gag, Beval, Universal 3-ring, Kimblewick) (22/454, 4.8%), horizontal shank (Extended double ring bit) (11/454, 2.4%) and Baucher (5/454, 1.1%). Double bridle was not the most used bit in any of the horses. The most common bit material was stainless steel (336/454, 74.0%), metal alloys or combinations of metals (68/454, 15.0%), plastic or rubber (40/454, 8.8%), and leather covered (10/454, 2.2%).

### Bit Size and Fit

**Figure 4:**
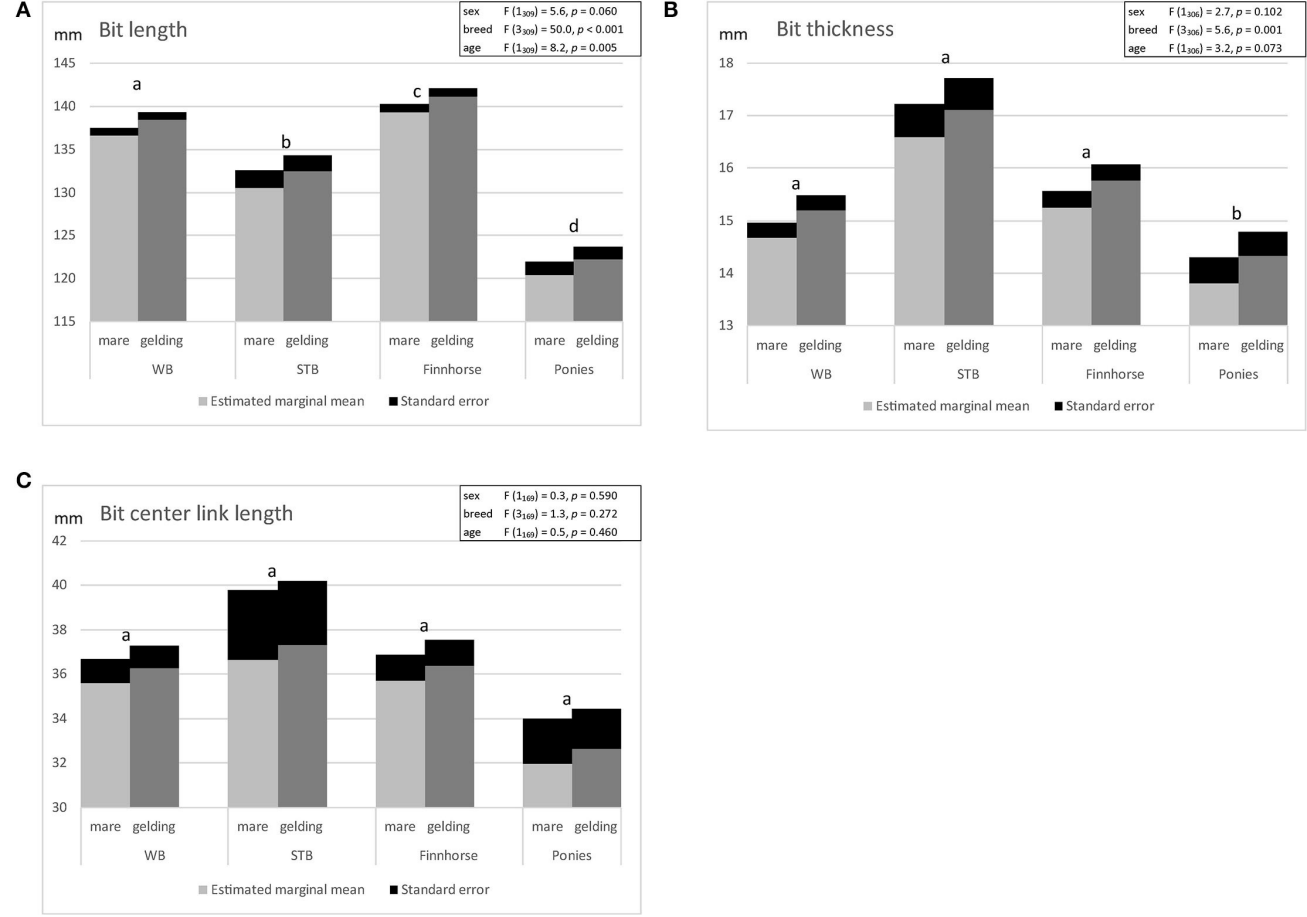
Estimated marginal means of linear regression model of bit mouthpiece size in mares and geldings of different breeds [Warmblood (WB), Standardbred (STB), Finnhorses, ponies] of adult horses and ponies ≥ 5 years: **(A)** bit length, **(B)** bit thickness, **(C)** bit center link length (significant differences between breeds marked by differing letters).

Definitions of the bit size and the correct bit fit are presented in Table 2. The variation in the bit mouthpiece size selected by the horse owners and the bit fit are presented in Table 3. The results of the linear regression model of bit mouthpiece size are presented in Figures 4A–C. Bit mouthpiece length correlated with age. Bit mouthpiece length and bit thickness were associated with breed. Center link length did not significantly correlate with age and was not associated with breed or sex. The linear regression model showed that breed and sex were 33.3% related to the selection of the bit mouthpiece length, 8.1% to bit thickness and 2.7% to center link length. In the selection of mouthpiece length, differences were found between all breeds, but in the selection of mouthpiece thickness, ponies differed from other breeds, and in the center link length selection, no difference was found between breeds (Figures 4A–C).

Horses and ponies had on average a 3.6 mm (SD 7.2 mm, *N* = 422) longer bit compared with mouth width, ranging from a 20 mm shorter bit to a 30 mm longer bit. Bit length fit in 313/422 (74.2%) horses. 109/422 (25.8%) horses had a bit length that did not fit, with a bit that was either too long (46/422, 10.9%) or too short (63/422, 14.9%).

**Figure 5:**
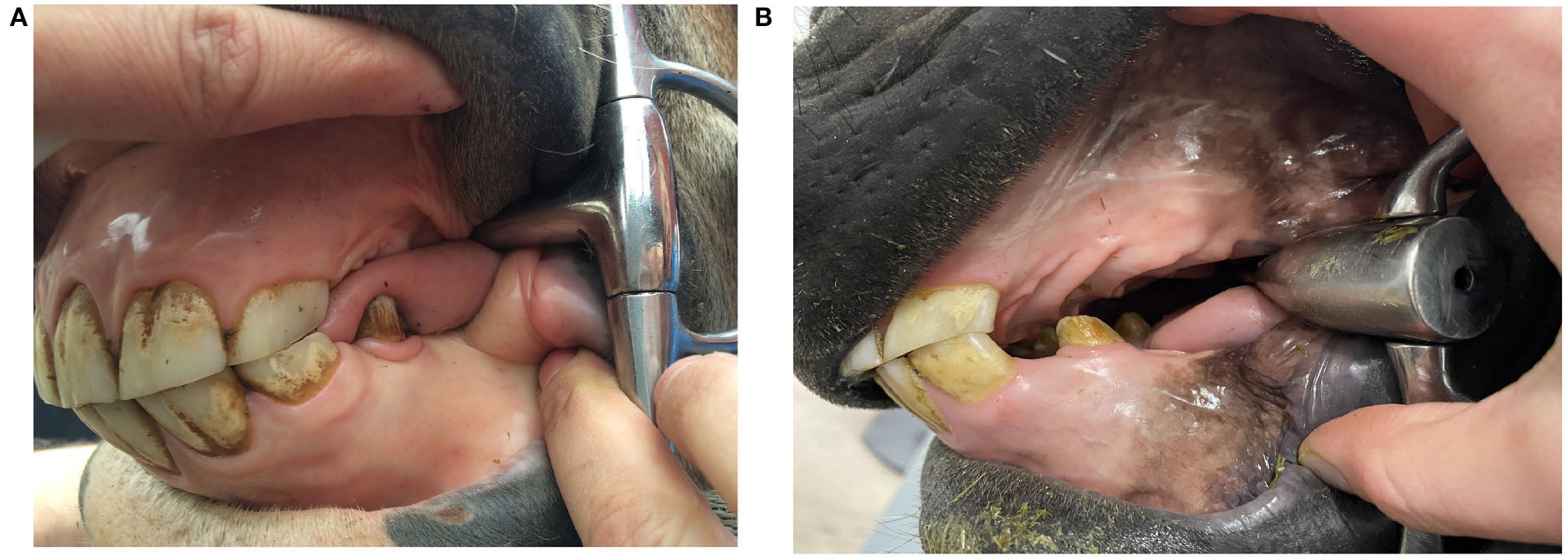
Bit thickness fit: **(A)** a bit that fits—tongue has enough space, **(B)** a bit that is too thick—too small space left for tongue, tongue retracted partly behind the bit.

The bit space ranged from 5 to 22 mm, with mean space 14.0 mm (SD 3.2 mm, *N* = 79). Fit of the bit thickness showed that, on average, horses had 0.3 mm (SD 3.5 mm, *N* = 43) of spare space left between the upper and lower jaw when the bit was in the mouth and tongue thickness was taken into account. Fit of the bit thickness ranged from −9 to 12 mm. When the value was negative, the bit compressed the tongue and was considered too thick (Figures 5A,B). Thus 31/43 (72.1%) horses had a bit that fit in terms of thickness and 12/43 (27.9%) had a bit that was too thick. The tongue space left with the used bit ranged from 0 to 36 mm (mean 13.7 mm, SD 4.7 mm, *N* = 419). Due to only a small number of horses having their tongue thickness measured, no linear regression model was conducted for bit fit variables.

Double-jointed or barrel bit center link length compared with lower jaw width ranged from −15 to 40 mm. The mean value was 5.9 mm (SD 8.5 mm, *N* = 215), meaning a 5.9 mm longer center link compared with lower jaw width. 151/215 (70.2%) horses had a central link length 1–40 mm longer than their lower jaw width, 34/215 (15.8%) had a central link length 1–15 mm shorter and 30/215 (14.013.9%) had a center link length similar to their lower jaw width.

A significant difference was found between mares and geldings in tongue space (*t*-test 2.68, *p* = 0.008) but not in other bit fit variables. Mares had on average 13.0 mm (SD 4.5 mm, *N* = 177) less tongue space compared with the 14.2 mm of geldings (SD 4.8 mm, *N* = 242).

## Discussion

### Oral Dimensions

As far as we know, this is the first study to measure oral dimensions in living sedated adult horses and ponies and to evaluate the fit of the bit by comparing oral dimensions with the currently used bit size. The oral dimensions of a sedated horse can be easily measured as part of routine oral examination and dental care. All oral dimensions were influenced by horse breed and sex. Mouth width and distance between upper and lower jaw were also influenced by age. The largest variation in oral dimensions was found between horse breeds. This supports previous findings that the largest variation in the skull dimensions between breeds is found in the nasal part of the skull (24, 25). In coldblood Finnhorses, the oral dimensions were greater than in other breeds, while they were smallest in ponies. In a previous study it was demonstrated that coldblooded horses have a relatively longer head and wider maxilla at the diastema compared with warmblood types (25). In a radiographic study with eight horses no relationship with wither height and oral dimensions was detected; however, the study population was small (four Warmblood and four Thoroughbred) (27). In our study, a positive association was found between oral dimensions, meaning that horses with a wider mouth also had a larger distance between the upper and lower jaw and a thicker tongue.

All oral dimensions of geldings were greater than those of mares, which may be explained by the effect of sex hormones on horse growth (28). The association of age with upper and lower jaw distance in adult horses could be explained by the hypsodont teeth and continuous eruption of cheek teeth in horses (29). However, tongue thickness was not related to age, which may suggest that with age, horses can gain more bit space, which is opposite to previous suggestion that horses may have less space for the bit as the horse ages due to sloping of the incisors (12). Moreover, molar teeth size and shape are related to age and breed and that the size of the molar tooth crown is established in young horses while body growth continues throughout life and is affected by sexual hormones (28). In the current study, 5-year-old horses were considered to have adult dentition, since the last cheek teeth (Triadan 11) erupt at age 3.5–4 years and the last deciduous incisors (Triadan 03) are shed at age 4–5 years (29). Although breed, age and sex explain part of the variation in the oral dimensions of adult horses and ponies, individual differences exist, which enhances the importance of measuring oral dimensions and evaluating bit fit regularly as the horse ages.

In our study, lower jaw width was narrower than reported in cadaver horses (20). In their study (20), the measuring point was not defined to be the bar center and may have been measured from the lateral sides of the bars instead. Since the lower jaw widens caudally and the position of the lip commissures in relation to the interdental space length is suggested to vary between horses (14, 20), the measuring point may vary in the rostro-caudal direction.

Soft tissues affect the oral dimensions and thus it is not known whether the difference found in the mouth width between Warmblood and Standardbred is due to the difference in the width of the bony maxilla or in the width of the lip commissures, or both. The measurements of the distance between the upper and lower jaw or the lower jaw width are to a lesser extent affected by the soft tissues, since bony surfaces are only covered by a thin mucosa (20). The tongue, however, may compress to a certain degree between caliper tips; thus tongue thickness measurement is affected by the muscular tone of the tongue, which may differ in sedated and non-sedated horses. This was the first study to measure tongue thickness with a plastic caliper and to show variation in tongue thickness as suggested in previous studies (7, 8). A thicker tongue in Finnhorses compared with other breeds may explain why coldblood horses are commonly considered to be less sensitive to the bit. A thicker tongue may give better cushioning over the sensitive bars of the lower jaw compared with horses with thinner tongue. On the other hand, a thick tongue leaves less space for the bit between the upper and lower jaw, potentially increasing the risk of pressure on the tongue if too thick a bit is used. Too high pressure may cause a horse to move its tongue, open its mouth or bite the bit between the cheek teeth to relieve or avoid pressure on the tongue (7, 8).

### Bit Length

It was common for the horse owners to use either too short, shorter than mouth width, or too long, over 10 mm longer than mouth width, bit mouthpiece. Horse breed and age appeared to affect the owner's selection of bit length. However, horse owners may be unaware of the relationship between oral dimensions and sex when selecting bit size. In our study, mares had narrower mouth width and less bit space. A bit that is too long moves sideways easily and may cause rubbing of the lip commissures (3, 12, 30). The joint of the bit mouthpiece can also move sideways and thus may cause a pinching “nutcracker effect” or pressure point on the side of the tongue and on the bar of the lower jaw (4, 5, 10, 30). With a jointed snaffle bit that is too long, the center link or joint moves rostrally in the mouth and this may cause the horse to lift the bit with its tongue or lift tongue on top of the bit (4). A bit that is too short can cause rubbing on the outer side of the lip commissures and press the cheeks inwards against the edges of the cheek teeth (4).

In the present study, the length of the bit mouthpiece was measured along the caudal surface of the bit taking into account the shape of the bit mouthpiece. Previously, it has been recommended to measure bit length in a straight line between the bit rings (4) ignoring the shape of the bit design. Generally, it is recommended to have a bit mouthpiece length equal to the mouth width (4) or not protruding more than 0.5 cm from each side (12). Fit of the bit length was defined accordingly in our study.

### Bit Thickness

The use of a bit that is too thick was common. The bit should fit between the upper and lower jaw when the horse's mouth is closed and leave enough space for the tongue (20). Selection of the bit thickness by the horse owners was only associated with horse breed. A difference in the selected bit thickness was only detected between Finnhorses and ponies, although differences in oral dimensions were seen between all breeds, sexes and ages. Commonly horses had room for a rather thin 14 mm bit. The use of a bit that is too thin or too thick has been recognized as a risk factor for oral trauma (17, 31). In the present study, the mean tongue space left with the bit was smaller than the mean tongue thickness, suggesting that, on average, horses had bits that were slightly too thick. A bit that is too thick can cause discomfort by compressing the tongue (4, 20), causing impeded blood flow and making the tongue “numb” (3) or restricting tongue movement (7). Horses can try to relieve the excessive pressure, for example, by opening the mouth, lifting the tongue over the bit, retracting or bulging the tongue or by grabbing the bit between the second premolar teeth (1, 7, 32). In a survey of the use of the noseband, a common reason given was to prevent the horse to lift tongue over the bit (33).

Historically the use of a thick bit has been justified by causing smaller pressure on the mouth (5). Pressure is inversely proportional to the contact area of the bit and mouth over which the force through the reins is distributed and thus the smaller the contact area, the higher the pressure (3, 9, 12, 34). However, previous studies have suggested that a thick mouthpiece in a small mouth can cause more discomfort than a thin bit (4, 20). Furthermore, it was demonstrated that horses ridden with a thinner bit showed signs of reduced stress compared with horses ridden with thicker bits (35). A tapering bit narrowing in a vertical dimension has a wider contact area with the lip commissures and a wide contact area with the tongue but leaves more space for the tongue between the upper and lower jaw (12). The tapering bit could be a solution for horses with a small bit space and thus difficulty in finding a bit that fits. The Fédération Equestre Internationale's dressage rules state that the minimum diameter of a mouthpiece is 12 mm for a curb bit, 10 mm for a bridoon bit and 12 mm for a snaffle bit in horses and 10 mm for a snaffle bit in ponies (19). However, the maximum bit thickness has not been stated. In the current study no horses had room for a bit that was over 22 mm thick; thus the use of bits over 22 mm thick is not recommended.

### Bit Type

Double-jointed snaffle was the most common bit type used. If the center link length is similar to the lower jaw width, it may cause pressure points or pinching of the edges of the tongue and the bars of the lower jaw, predisposing to injury (6, 10, 12, 27). Horse can try to relief the pressure on the tongue by moving the tongue or lifting the over the bit (9, 10). In the current study, 13.9% of horses had a center link length similar to the lower jaw width. It has been suggested that an extra link reduces the pressure on the bars compared with a single-jointed snaffle, the short center link causing more pressure on the bars of the lower jaw and the longer center link more on the tongue (36). A bit is mobile and the center link can change position and orientation inside the mouth with rein tension (5, 6, 10, 34). Based on the radiographic study of the bit position within the horse's oral cavity, it is recommended that the center link should be short to avoid placing the joints directly over the bars of the lower jaw (6). Large variability in the surface morphology of the bars of the lower jaw has been noted, with some horses having rounded and others sharp-edged bars, which may predispose to injury (18, 37). The orientation of the center link of the Bristol/French-link-type double-jointed bit affects the severity of the bit, a narrow surface on top of the tongue causing more pressure (5, 10). Double jointed bits have high variety of mouthpiece design and tongue angle, some having sharp mouthpiece features causing a region of high pressure onto the tongue (10). Center link length and bit–mouth contact area as potential risk factors for tongue and lower jaw bar trauma remains to be studied. Trauma of the bars of the lower jaw has been reported in harness racing horses (16), eventing horses (17), competing Icelandic horses (13) and polo ponies (14).

Horse owners seem to be unaware of the variation in the lower jaw width by horse sex and breed when selecting a center link length and type, since the selected center link length was not associated with breed or sex. In the literature, no evidence-based guidelines have been given on fit of the center link of the double-jointed or barrel bit.

## Conclusion

This study showed that oral dimensions vary by age, sex and breed in adult horses and ponies. Horses commonly had bits that did not fit in terms of the bit size in relation to oral dimensions. Horses have on average room for a 14 mm thick bit without causing compression of the tongue. Measuring oral dimensions as part of routine dental examination aids in choosing a bit mouthpiece size that fits in order to avoid discomfort. It is recommended to check bit fit regularly as the horse ages.

## Data Availability Statement

The original contributions presented in the study are included in the article/Supplementary Material, further inquiries can be directed to the corresponding author/s.

## Ethics Statement

The animal study was reviewed and approved by University of Helsinki Viikki Campus Research Ethics Committee. Written informed consent for participation was not obtained from the owners because study design was retrospective and performed as part of horses routine dental care.

## Author Contributions

MA contributed to the study design, data collection, statistical analyses, preparation of the manuscript and takes responsibility for the integrity of the data, and the accuracy of the data analysis. AV and MR contributed to the statistical analyses, interpretation of the results, and manuscript preparation. All authors have read and approved the final manuscript.

## Conflict of Interest

The authors declare that the research was conducted in the absence of any commercial or financial relationships that could be construed as a potential conflict of interest.

## Publisher's Note

All claims expressed in this article are solely those of the authors and do not necessarily represent those of their affiliated organizations, or those of the publisher, the editors and the reviewers. Any product that may be evaluated in this article, or claim that may be made by its manufacturer, is not guaranteed or endorsed by the publisher.
