# Supplementary material for: Oral Dimensions Related to Bit Size in Adult Horses and Ponies
Source: Front Vet Sci. 2022 May 12;9:879048. doi: 10.3389/fvets.2022.879048 (PMC9133790; doi:10.3389/fvets.2022.879048)
Supplement: Supplementary file 1 [file Table_1.docx]

**SUPPLEMENTARY MATERIAL**

**Table S1. Correlations between measured oral dimensions in mares and geldings of different breeds of adult horses and ponies ≥ 5 years (significant correlations are marked in bold)**

|  | | Mouth width | Distance between upper and lower jaw | Lower jaw width | Tongue thickness |
| --- | --- | --- | --- | --- | --- |
| Mouth width | Pearson correlation | 1 | **0.447** | **0.444** | **0.375** |
|  | Significance (two-tailed) |  | 0.000 | 0.000 | 0.001 |
|  | N | 554 | 554 | 508 | 79 |
| Distance between upper and lower jaw | Pearson correlation | **0.447** | 1 | **0.250** | **0.545** |
|  | Significance (two-tailed) | 0.000 |  | 0.000 | 0.000 |
|  | N | 554 | 554 | 508 | 79 |
| Lower jaw width | Pearson correlation | **0.444** | **0.250** | 1 | 0.113 |
|  | Significance (two-tailed) | 0.000 | 0.000 |  | 0.322 |
|  | N | 508 | 508 | 508 | 79 |
| Tongue thickness | Pearson correlation | **0.375** | **0.545** | 0.113 | 1 |
|  | Significance (two-tailed) | 0.001 | 0.000 | 0.322 |  |
|  | N | 79 | 79 | 79 | 79 |
